# Transcriptome Analysis of Long Non-coding RNAs and Genes Encoding Paraspeckle Proteins During Human Ovarian Follicle Development

**DOI:** 10.3389/fcell.2018.00078

**Published:** 2018-07-24

**Authors:** Emil H. Ernst, Julie Nielsen, Malene B. Ipsen, Palle Villesen, Karin Lykke-Hartmann

**Affiliations:** ^1^Department of Biomedicine, Aarhus University, Aarhus, Denmark; ^2^Bioinformatic Research Centre, Aarhus University, Aarhus, Denmark; ^3^Department of Clinical Medicine, Aarhus University, Aarhus, Denmark; ^4^Department of Clinical Genetics, Aarhus University Hospital, Aarhus, Denmark

**Keywords:** human follicle, lncRNA, paraspeckle, fertility, treatment

## Abstract

Emerging evidence indicated that many long non-coding (lnc)RNAs function in multiple biological processes and dysregulation of their expression can cause diseases. Most regulatory lncRNAs interact with biological macromolecules such as DNA, RNA, and protein. LncRNAs regulate gene expression through epigenetic modification, transcription, and posttranscription, through DNA methylation, histone modification, and chromatin remodeling. Interestingly, differential lncRNA expression profiles in human oocytes and cumulus cells was recently assessed, however, lncRNAs in human follicle development has not previously been described. In this study, transcriptome dynamics in human primordial, primary and small antral follicles were interrogated and revealed information of lncRNA genes. It is known that some lncRNAs form a complex with paraspeckle proteins and therefore, we extended our transcriptional analysis to include genes encoding paraspeckle proteins. Primordial, primary follicles and small antral follicles was isolated using laser capture micro-dissection from ovarian tissue donated by three women having ovarian tissue cryopreserved before chemotherapy. After RN sequencing, a bioinformatic class comparison was performed and primordial, primary and small antral follicles were found to express several lncRNA and genes encoding paraspeckle proteins. Of particular interest, we detected the lncRNAs *XIST, NEAT1, NEAT2 (MALAT1)*, and *GAS5*. Moreover, we noted a high expression of *FUS, TAF15*, and *EWS* components of the paraspeckles, proteins that belong to the FET (previously TET) family of RNA-binding proteins and are implicated in central cellular processes such as regulation of gene expression, maintenance of genomic integrity, and mRNA/microRNA processing. We also interrogated the intra-ovarian localization of the FUS, TAF15, and EWS proteins using immunofluorescence. The presence and the dynamics of genes that encode lncRNA and paraspeckle proteins may suggest that these may mediate functions in the cyclic recruitment and differentiation of human follicles and could participate in biological processes known to be associated with lncRNAs and paraspeckle proteins, such as gene expression control, scaffold formation and epigenetic control through human follicle development. This comprehensive transcriptome analysis of lncRNAs and genes encoding paraspeckle proteins expressed in human follicles could potentially provide biomarkers of oocyte quality for the development of non-invasive tests to identify embryos with high developmental potential.

## Introduction

The nuclei of mammalian cells are highly organized and composed of distinct subnuclear structures termed nuclear bodies (Naganuma and Hirose, 2013; Yamazaki and Hirose, 2015). Paraspeckles are mammalian-specific sub-nuclear bodies built on long, non-protein-coding RNA (lncRNA), *NEAT1* (nuclear-enriched abundant transcript 1), which assembles various protein components, including RNA-binding proteins of the DBHS (Drosophila behavior and human splicing) family. Paraspeckles have been proposed to control of several biological processes, such as stress responses, gene expression, and cellular differentiation. Human follicle development represents a continuous cyclic process throughout the reproductive lifespan of a woman and encompasses both cell growth and differentiation. Paraspeckles are among the most recently identified nuclear bodies and were first described in 2002 (Fox et al., 2002; Bond and Fox, 2009). The generation of paraspeckle sub-nuclear compartments has been extensively described (Naganuma and Hirose, 2013; Yamazaki and Hirose, 2015). Paraspeckles are sensitive to RNAse treatment, suggesting that their structures depend on RNAs for maintenance (Fox et al., 2002, 2005). Later the lncRNA *NEAT1* was shown to be essential for paraspeckle formation, as a knockdown of the *NEAT1* lncRNA function caused a disintegration of paraspeckles (Chen and Carmichael, 2009; Clemson et al., 2009; Sasaki et al., 2009; Sunwoo et al., 2009). Paraspeckle formation proceeds in conjunction with *NEAT1* lncRNA biogenesis and involves the cooperation of multiple paraspeckle-localized RNA-binding proteins (Naganuma and Hirose, 2013; Yamazaki and Hirose, 2015). Currently about 40 proteins are known to assemble in paraspeckles (Naganuma et al., 2012). Paraspeckle proteins include DBHS (Drosophila melanogaster behavior, human splicing) proteins, PSPC1 (paraspeckle component 1), NONO (non-POU domain-containing octamer-binding), and SFPQ [splicing factor, proline- and glutamine-rich (also known as PSF (PTB-associated splicing factor)], RNA binding motif (RBM) 14, and CPSF6 (cleavage and polyadenylation specific factor 6) [Reviewed in (Yamazaki and Hirose, 2015)]. Many paraspeckle proteins are RNA binding proteins that contain an RNA recognition motif (RRM), a KH (hnRNP K homology) domain, a RGG (glycine-arginine-rich) box, or a zinc finger motif as the RNA-binding domain. The paraspeckle proteins NONO, SFPQ, RBM14, EWS, FUS, TAF15, and TDP-43 are RNA binding proteins that mediate transcription and RNA processing (Auboeuf et al., 2005).

The paraspeckle-localized FET family of RNA-binding proteins (Bertolotti et al., 1996) consists of FUS (TLS) (Crozat et al., 1993), EWS (Delattre et al., 1992), and TAF15 (TAFII68, TAF2N, RBP56) (Crozat et al., 1993). The proteins are structurally similar and contain a number of evolutionary conserved areas such as the RRM motif, the SYGQ-rich domain, a G rich domain, a RanBP2-type zinc finger motif, and the C-terminal RGG domain (Morohoshi et al., 1998; Guipaud et al., 2006; Nguyen et al., 2011; Chau et al., 2016). The FUS, EWS, and TAF15 proteins bind RNA as well as DNA and have both unique and overlapping functions. The human FET proteins are associated with transcription (Law et al., 2006), RNA splicing, microRNA (miRNA) processing, RNA transport, and the signaling and maintenance of genomic integrity (Schwartz et al., 2015).

Several paraspeckle proteins are disease-related. For instance, NONO, SFPQ, CPSF6, EWS, FUS, TAF15, DAZAP1, RBM3, SS18L1, WT1, BCL6, BCL11A, ZNF4444, and HNRNPH1 are implicated in various types of cancer (reviewed in Yamazaki and Hirose, 2015). Some paraspeckle proteins, such as TDP-13, FUS, EWS, TAF15, HNRNPA1, SS18L1, and SFPQ have been associated with neuro-degenerative diseases, such as amyotrophic lateral sclerosis (ALS) and frontotemporal dementia (FTD) (Svetoni et al., 2016).

Paraspeckles have been described as nuclear sponges sequestering transcription factors and/or RNA-binding proteins such as lncRNAs. They are dynamic structures changing in size in response to ever changing cellular challenges/environment (Yamazaki and Hirose, 2015).

In addition to *NEAT1*, a number of lncRNAs localize to different subcellular compartments (Chen and Carmichael, 2010). *MALAT1* (*NEAT2* in human) is transcribed downstream of the *NEAT1* gene and is found specifically associated with splicing speckles (Hutchinson et al., 2007). Moreover, lncRNA have also been implicated in stem cell pluripotency and in differentiation in mice (Dinger et al., 2008). Furthermore, roles for ncRNAs in cell fate decision have been explored (Ambasudhan et al., 2011; Yoo et al., 2011; Kurian et al., 2013).

Interestingly, lncRNAs have been shown to act as chromatin modifiers (Mercer et al., 2009) and potent regulators of histone methylation (Yamazaki and Hirose, 2015), including chromatin structure modeling and the integrity of subcellular compartments (Chen and Carmichael, 2010; Wang and Chang, 2011; Wang et al., 2011; Wapinski and Chang, 2011; Yan et al., 2012; Backofen and Vogel, 2014; Joh et al., 2014; Peschansky and Wahlestedt, 2014; Liu and Pan, 2015). A previous study showed that some human lncRNAs were bound to the polycomb repressive complex 2 (PRC2) and other chromatin-modifying complexes (Khalil et al., 2009).

Several lncRNA, including *Xist, Tsix*, and *Xite* contribute to X chromosome inactivation, the process of ensuring dosage regulation of X chromosome-expressed genes (Chow and Heard, 2009; Leeb et al., 2009) in a complex and highly controlled manner (Zhao et al., 2008). Furthermore, *Xist* transcription is required for maintenance of X-chromosome inactivation (Penny et al., 1996). Interestingly, another lncRNA, *RepA*, the reassembling part of the 5′UTR sequence of *Xist*, was found to associate indirectly with PRC2 (Zhao et al., 2008). The recruitment of PRC2 by *RepA* happens in competition with lncRNA *Tsix*, which acts as an antisense toward *Xist*, and the binding of *RepA* to PRC2 is inhibited by *Tsix*, and thus competes with *RepA* (Zhao et al., 2008).

In support of the developmental roles of lncRNA and paraspeckles, *Neat1* knockout (KO) mice fail to become pregnant despite normal ovulation, which was found to be a caused by corpus luteum dysfunction and concomitant low progesterone (Nakagawa et al., 2014).

The developmental capacity of the matured oocyte for generating viable offspring is determined throughout follicle development in the ovary. The integrity of the oocytes is essential in maintaining the reproductive potential of the female. Pre-ovulatory oocyte maturation is a complex process resulting from multiple interactions between the oocyte and the surrounding follicular cells (Carabatsos et al., 2000; Adhikari and Liu, 2009; Binelli and Murphy, 2010; Reddy et al., 2010; Bonnet et al., 2013). The transition from primordial to primary follicle is a key first step event in follicle development, in which the primordial follicle is believed to have escaped the resting phase and has entered the follicular growth phase (Zuccotti et al., 2011). Subsequently, the cohort of follicles must remain activated in order to enter the secondary follicle stage, and a few continue to mature to the tertiary and antral follicle stages (McGee and Hsueh, 2000). Tertiary and antral follicles are characterized by the presence of a cavity known as the antrum, and have both granulosa and theca cells present. Tertiary follicles have an extensive network of gap junctions that permits the transfer of nutrients and regulatory signals between the oocyte and the granulosa cells (Espey, 1994). Only a small fraction of the ovarian follicles present in a fetal ovary will reach ovulation (Markström et al., 2002). Identifying the factors controlling follicle development may provide a basis for the fundamental mechanisms that regulate follicle activation and could potentially lead to new therapeutics in female reproduction as well as improvements in reproductive health and productivity in women of advanced maternal age (Baird et al., 2005). As paraspeckles and the regulatory molecules sequestered within them have been shown to be of importance in development, gene expression, and epigenetic control, these nuclear structures may prove essential in human fertility and infertility.

So far, only limited reports of the potential regulatory impact of short ncRNA in follicle development exist and our knowledge of the involvement of lncRNAs in human follicle development is almost non-existent (Wilhelmm and Bernard, 2016).

Therefore, in this study, the presence of lncRNAs were interrogated bioinformatically using RNA sequencing data representative of selected stages in human follicle development. We previously developed a method for isolating pure populations of oocytes from human primordial, intermediate and primary follicles using laser capture micro-dissection microscopy (Markholt et al., 2012). From these transcriptome data (Ernst et al., 2017, 2018), *in silico* extraction of data for lncRNAs. We identified the presence of the paraspeckle forming lncRNAs *NEAT1* and *NEAT2* as well as several other lncRNAs, such as *XIST*. As the discovery of *NEAT1* and *NEAT2* in early ovarian follicles suggested the presence of paraspeckle proteins, we further asked if genes encoding these proteins would also be present during human ovarian follicle development. We found the transcripts encoding the well-characterized FUS, EWS, and TAF15 highly expressed during early ovarian follicle development. We further employed immunohistochemistry in human ovary tissue to explore the presence and intraovarian localization of FUS, EWS, and TAF15 proteins to be present.

In summary, we identified the presence of several lncRNAs and genes encoding paraspeckle proteins not previously reported for human ovarian follicle development. This may hint that the functions of lncRNAs and paraspeckle proteins could indeed be relevant to oocyte physiology and development.

## Materials and methods

### Procurement of human ovarian cortex and isolation of oocytes and supportive somatic cells

We procured human ovarian cortex tissue from the Danish Cryopreservation Programme offering cryopreservation as means of fertility preservation prior to gonadotoxic chemotherapy (Rosendahl et al., 2011). Oocyte samples were obtained from ovarian cortical tissue procured from three patients who underwent unilateral oophorectomy prior to gonadotoxic treatment for a malignant disease (unrelated to any ovarian malignancies). Patients were normo-ovulatory, with normal reproductive hormones, and not received ovarian stimulation with exogenous gonadotropins. All methods were carried out in accordance with relevant guidelines and regulations, and The Central Denmark Region Committees on Biomedical Research Ethics and the Danish Data Protection Agency approved the study. Written informed consent was obtained from all participants before inclusion. Patients consented to the research conducted. In subjects undergoing oophorectomy, a small piece of the ovarian cortex is used for evaluating the ovarian reserve, and for research purposes (Danish Scientific Ethical Committee Approval Number: KF 299017 and J/KF/01/170/99) (Schmidt et al., 2003).

### Laser capture micro-dissection (LCM)

**Figure 1:**
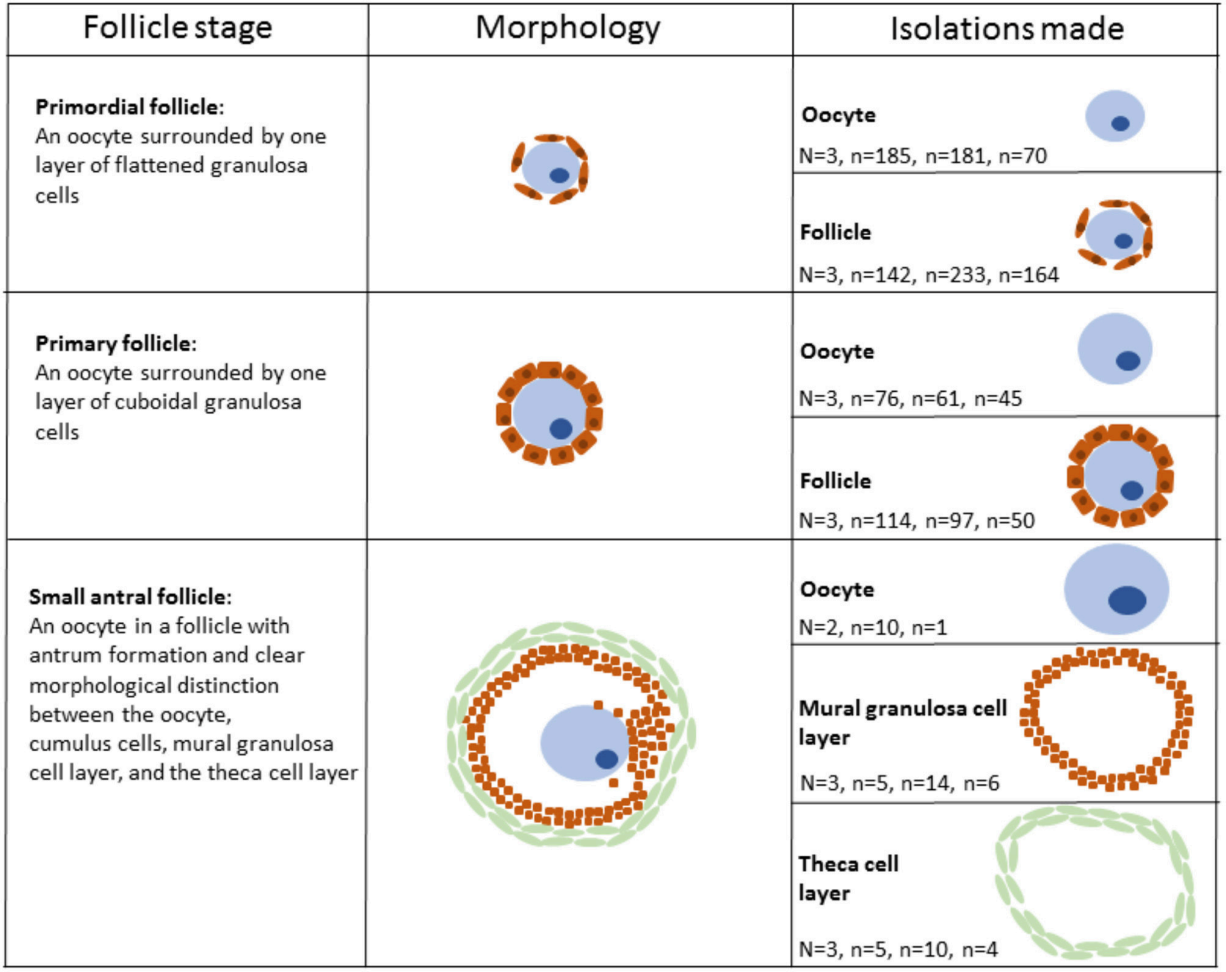
Schematic illustration of human follicular cells isolated using laser capture microdissection. Please note that the aspect ratio is arbitrary.

The LCM procedure to isolate staged oocytes and follicles was performed as previously described (Markholt et al., 2012; Ernst et al., 2017, 2018). Briefly, the ovarian cortical fragments, which had a size of 2 × 2 × 1 mm, were thawed and fixed by direct immersion into 4% paraformaldehyde (PFA) at 4°C for 4 h followed by dehydration and embedding in paraffin. Paraffin blocks were stored at −80°C until use. The blocks were cut into 15 μm thick sections on a microtome (Leica Microsystems, Wetzlar, Germany). Diethylpyrocarbonate (DEPC)-treated water was used in the microtome bath to avoid RNA degradation. The sections were mounted on RNase-free membrane glass slides (Molecular Devices, Sunnyvale, CA, USA) and immediately processed. Consecutively, the slides were de-paraffinized, stained, and dehydrated immediately before micro-dissection: Xylene (VWR—Bieog Berntsen, Herlev, Denmark) (5 min), 99.9% ethanol (Merck, Darmstadt, Germany) (5 min), 99.9% ethanol (5 min), 96% ethanol (5 min), 70% ethanol (5 min), DEPC-treated water (5 min), hematoxylin (Merck, Darmstadt, Germany) (5 min), DEPC-treated water (immersion), 70% ethanol (30 s), 96% ethanol (30 s), 99.9% ethanol (30 s), 99.9% ethanol (30 s), xylene (1 min), and xylene (5 min). All solutions were prepared with DEPC-treated water. LCM was performed using the Veritas™ Microdissection Instrument Model 704 (ArcturusXT™, Molecular Devices, Applied Biosystems®, Life Technologies, Foster City, CA, U.S.A.). The cells were isolated based on morphological appearance. Primordial oocytes were defined as an oocyte surrounded by 3–5 flattened pre-granulosa cells, and primary oocytes were defined as an oocyte surrounded by one layer of cuboidal granulosa cells. Antral follicles were defined as a follicle with an antral cavity. For the antral follicle to be eligible for isolation, we should be able to morphologically differentiate between the oocyte, the mural granulosa cells and the theca cell layer. In the antral stage the large size of the different compartments enabled us to isolate each compartment individually. An outline surrounding the cell(s) of interest was marked and subsequently cut using the ultraviolet laser. Following this the use of membrane glass slides (Arcturus® PEN Membrane Glass Slides, Applied Biosystems, Life Technologies, Foster City, CA, U.S.A.) enabled us to lift the isolate onto a sterile cap (Arcturus® CapSure® HS LCM Caps, Applied Biosystems, Life Technologies, Foster City, CA, U.S.A.) using infrared pulses. Isolated cells were inspected on the cap to ensure that no contamination from surrounding unwanted cells was present. From each of the three patients, several isolations were made (Table 1, Figure 1). RNA isolation, library preparation and sequencing, mapping and statistical analysis and bioinformatics were performed as described (Ernst et al., 2017, 2018).

**Table 1:** Numbers of oocytes, follicles, and other somatic cells analyzed in RNA–seq. in three different patients.

**Cell type and Follicular Stages**	**No. of laser-collected cells from three patients respectively**
Oocytes from primordial follicles	*N* = 3, *n* = 185, *n* = 181, *n* = 70
Primordial follicles	*N* = 3, *n* = 142, *n* = 233, *n* = 164
Oocytes from primary follicles	*N* = 3, *n* = 76, *n* = 61, *n* = 45
Primary follicles	*N* = 3, *n* = 114, *n* = 97, *n* = 50
Mural granulosa cell layers from small antral follicles	*N* = 3, *n* = 5, *n* = 14, *n* = 6
Theca cell layers from small antral follicles	*N* = 3, *n* = 5, *n* = 10, *n* = 4
Oocytes from small antral follicles^*1^	*N* = 2, *n* = 10, *n* = 1

*1 *Based on duplicate samples*.

### Library preparation and sequencing

RNA was extracted from the LCM-derived samples, converted to cDNA and subjected to linear amplification [Ovation® RNA-Seq System V2 kit (NuGen Inc., San Carlos, CA, U.S.A.)]. RNA-seq libraries (constructed from the output cDNA using Illumina TruSeq DNA Sample and Preparation kit (Illumina, San Diego, CA, USA, according to AROS Applied Biotechnology, now Eurofins (https://www.eurofinsgenomics.eu/). Integrity of libraries was verified on library yield via KAPA qPCR measurement, and Agilent Bioanalyzer 2100 peak size with a RNA 6000 Nano Lab Chip (Agilent Technologies, Santa Clare, CA, U.S.A.) during different library preparation stages. Sequencing was performed on an Illumina HiSeq2000 platform (Illumina Inc., San Diego, CA, U.S.A.) with 5 random samples per lane (AROS Applied Biotechnology).

### Mapping and statistical analysis

Using Tophat (2.0.4), and Cufflinks (2.0.2) BAM files were generated to create a list of expressed transcripts in the samples. BWA (0.6.2) was subsequently used to map all readings to the human reference genome (hg19) using the transcript list as a filter so only readings mapping to RefSeq exons [incl. non-coding RNA, and mitochondrial RNA) overlapping with expressed transcripts were used. Expression of each gene in a given sample was normalized and transformed to a measurement of log2 (counts per million (CPM)]. On the basis of log2 (CPM), fragments per kilobase of exon per million fragments mapped (FPKM) values were calculated, and further filtered using custom analysis in R [R Development Core Team. R: A language and environment for statistical computing. R Foundation for Statistical Computing, Vienna, Austria. URL http://www.R-project.org] (R Core Team, 2012).

### Output from statistical analysis

The mean FPKM value for all ncRNA transcripts was calculated using a one-sample *t*-test on FPKM values for each identified transcript from patient triplicates (two for oocytes from small antral follicles) (Resource Data) (http://users-birc.au.dk/biopv/published_data/ernst_et_al_ncRNAs_2018/).

Cell Specific Consistently Expressed Genes (CSCEG) were defined as one-sample *t*-test *p*-value < 0.05 [(Resource Data), in gray]. *In silico* merging of transcriptomes from three patients was performed to account for biological variance. The transcripts in the CSCEG list were ranked based on *p*-value, with low *p*-value indicating a high degree of consistency in FPKM gene expression level between patients for the given isolate type. Full lists of ncRNAs detected in human follicle development is availiable (Supplementary Table 1).

Furthermore, we generated a list of all known paraspeckle proteins based on annotated protein-coding RNAs detected (Table 2) and non-coding RNAs (Table 3). Some of these data (oocytes and granulosa cells from primordial and primary follicles) has previously been published with a different focus in a global expression profile study (Ernst et al., 2017, 2018).

**Table 2:** Expression of paraspeckle-protein encoding mRNAs in human follicle development.

**Ensemble**	**Symbol**	**Oocytes from primordial follicles, FPKM mean**	***t*-test (*p*-value)**	**Primordial follicles, FPKM mean**	***t*-test (p-value)**
**PRIMORDIAL FOLLICLES**					
ENSG00000126705	AHDC1	ND	ND	0.195	0.423
ENSG00000011243	AKAP8L	2.094	0.366	0.347	0.423
ENSG00000140488	CELF6	ND	ND	ND	ND
ENSG00000099622	CIRBP	1.826	0.053	2.486	0.059
ENSG00000111605	CPSF6	3.718	0.033	4.289	0.064
ENSG00000149532	CPSF7	2.089	0.351	2.739	0.003
ENSG00000071626	DAZAP1	1.817	0.408	1.368	0.192
ENSG00000064195	DLX3	ND	ND	ND	ND
ENSG00000182944	EWS/EWSR1^*1^	5.765	0.073	6.132	0.173
ENSG00000119812	FAM98A	1.727	0.235	2.013	0.188
ENSG00000182263	FIGN	0.284	0.187	2.842	0.184
ENSG00000089280	FUS^*1^	3.150	0.149	3.150	0.146
ENSG00000139675	HNRNPA1L2	0.029	0.423	0.060	0.423
ENSG00000169813	HNRNPF	2.550	0.028	3.836	0.085
ENSG00000169045	HNRNPH1	3.410	0.017	6.310	0.002
ENSG00000096746	HNRNPH3	3.177	0.171	3.178	0.199
ENSG00000165119	HNRNPK	5.365	0.068	5.511	0.060
ENSG00000125944	HNRNPR	4.346	0.107	3.778	0.127
ENSG00000105323	HNRNPUL1	4.041	0.005	6.086	0.007
ENSG00000176624	MEX3C*1	1.365	0.204	3.570	0.160
ENSG00000147140	NONO*1	2.160	0.189	4.367	0.089
ENSG00000167005	NUDT21	4.365	0.155	5.401	0.065
ENSG00000121390	PSPC1	1.709	0.274	3.114	0.189
ENSG00000102317	RBM3	0.475	0.238	0.586	0.423
ENSG00000268489	RBM3	ND	ND	ND	ND
ENSG00000173914	RBM4B	1.878	0.359	1.489	0.400
ENSG00000076053	RBM7	1.519	0.330	1.191	0.222
ENSG00000244462	RBM12	4.391	0.106	4.400	0.112
ENSG00000239306	RBM14	1.072	0.207	1.164	0.394
ENSG00000147274	RBMX	4.218	0.117	3.981	0.122
ENSG00000020633	RUNX3	ND	ND	0.054	0.423
ENSG00000116560	SFPQ	4.459	0.025	5.770	0.071
ENSG00000188529	SRSF10	1.465	0.373	3.245	0.109
ENSG00000184402	SS18L1	0.083	0.423	1.002	0.213
ENSG00000172660	TAF15^*1^	1.303	0.172	2.055	0.226
ENSG00000143569	UBAP2L	7.856	0.006	7.315	0.006
ENSG00000188177	ZC3H6	6.564	0.002	2.015	0.365
		**Oocytes from primary follicles**		**Primary follicles**	
**Ensemble**	Symbol^*1^	**FPKM mean**	***t*****-test (*****p*****-value)**	**FPKM mean**	***t*****-test (*****p*****-value)**
**PRIMARY FOLLICLES**					
ENSG00000198026	ZNF335	ND	ND	1.216	0.423
ENSG00000120948	TARDBP	5.408	0.131	4.119	0.112
ENSG00000126705	*AHDC1*	0.883	0.423	ND	ND
ENSG00000011243	*AKAP8L*	ND	ND	ND	ND
ENSG00000140488	*CELF6*	1.933	0.186	4.296	0.196
ENSG00000099622	*CIRBP*	2.339	0.359	1.864	0.423
ENSG00000111605	*CPSF6*	1.331	0.246	3.621	0.189
ENSG00000149532	*CPSF7*	0.208	0.423	1.386	0.399
ENSG00000071626	*DAZAP1*	ND	ND	ND	ND
ENSG00000064195	*DLX3*	4.564	0.087	5.184	0.185
ENSG00000182944	EWS/EWSR1^*2^^,^^3^	0.835	0.423	1.826	0.409
ENSG00000119812	*FAM98A*	2.007	0.423	1.189	0.401
ENSG00000182263	*FIGN*	2.666	0.066	2.451	0.268
ENSG00000089280	FUS^*2^^,^^3^	0.158	0.204	0.439	0.423
ENSG00000139675	*HNRNPA1L2*	3.494	0.280	3.308	0.147
ENSG00000169813	*HNRNPF*	2.572	0.176	4.849	0.118
ENSG00000169045	*HNRNPH1*	6.586	0.022	2.636	0.287
ENSG00000096746	*HNRNPH3*	4.526	0.097	3.360	0.217
ENSG00000165119	*HNRNPK*	0.554	0.244	3.590	0.195
ENSG00000125944	*HNRNPR*	2.285	0.110	2.451	0.185
ENSG00000105323	*HNRNPUL1*	1.790	0.336	3.198	0.184
ENSG00000176624	MEX3C^*1^	3.151	0.046	3.493	0.167
ENSG00000147140	NONO^*1^	4.165	0.201	5.300	0.047
ENSG00000167005	*NUDT21*	1.994	0.312	3.174	0.199
ENSG00000121390	*PSPC1*	1.712	0.133	1.077	0.374
ENSG00000102317	*RBM3*	ND	ND	ND	ND
ENSG00000268489	*RBM3*	2.873	0.253	1.890	0.409
ENSG00000173914	*RBM4B*	0.654	0.423	0.161	0.259
ENSG00000076053	*RBM7*	5.893	0.057	5.262	0.177
ENSG00000244462	*RBM12*	0.952	0.423	0.716	0.353
ENSG00000239306	*RBM14*	4.337	0.019	3.784	0.175
ENSG00000147274	*RBMX*	ND	ND	ND	ND
ENSG00000020633	*RUNX3*	5.408	0.027	4.258	0.206
ENSG00000116560	*SFPQ*	3.097	0.160	4.403	0.176
ENSG00000188529	*SRSF10*	0.564	0.423	0.5280	0.374
ENSG00000184402	*SS18L1*	0.411	0.423	2.030	0.306
ENSG00000172660	TAF15^*1^	7.527	0.013	7.630	0.002
ENSG00000143569	*UBAP2L*	4.010	0.184	2.057	0.411
ENSG00000188177	*ZC3H6*	ND	ND	0.911	0.423
ENSG00000198026	*ZNF335*	1.576	0.378	2.515	0.393
ENSG00000120948	*TARDBP*	ND	ND	ND	ND
		**Mural garnulosa cell layer**		**Theca cell layer**	
**Ensemble**	**Symbol**	**FPKM mean**	***t*****-test (*****p*****-value)**	**FPKM mean**	***t*****-test (*****p*****-value)**
**SMALL ANTRAL FOLLICLES**					
ENSG00000126705	*AHDC1*	1.967	0.333	0.955	0.134
ENSG00000011243	*AKAP8L*	1.100	0.132	2.477	0.103
ENSG00000140488	*CELF6*	ND	ND	0.181	0.423
ENSG00000099622	*CIRBP*	3.468	0.017	4.523	0.021
ENSG00000111605	*CPSF6*	4.481	0.022	3.007	0.039
ENSG00000149532	*CPSF7*	4.775	0.001	1.925	0.075
ENSG00000071626	*DAZAP1*	1.899	0.063	1.419	0.045
ENSG00000064195	*DLX3*	ND	ND	ND	ND
ENSG00000182944	***EWS/EWSR1***	8.561	0.001	6.354	0.009
ENSG00000119812	*FAM98A*	3.164	0.057	4.066	0.025
ENSG00000182263	*FIGN*	3.326	0.018	3.210	0.023
ENSG00000089280	***FUS***	4.624	0.003	3.323	0.077
ENSG00000139675	*HNRNPA1L2*	1.234	0.136	1.193	0.263
ENSG00000169813	*HNRNPF*	3.712	0.004	4.046	0.001
ENSG00000169045	*HNRNPH1*	6.458	0.011	6.056	0.004
ENSG00000096746	*HNRNPH3*	4.316	0.033	3.635	0.014
ENSG00000165119	*HNRNPK*	5.862	0.004	5.249	1.7237E-05
ENSG00000125944	*HNRNPR*	5.096	0.011	4.504	0.014
ENSG00000105323	*HNRNPUL1*	6.769	0.002	5.058	0.049
ENSG00000176624	*MEX3C*	5.705	0.006	2.639	0.036
ENSG00000147140	*NONO*	6.395	0.002	5.664	0.027
ENSG00000167005	*NUDT21*	5.327	0.015	3.735	0.131
ENSG00000121390	*PSPC1*	2.929	0.086	4.015	0.003
ENSG00000102317	*RBM3*	3.057	0.104	4.013	0.033
ENSG00000268489	*RBM3*	ND	ND	ND	ND
ENSG00000173914	*RBM4B*	2.125	0.140	1.097	0.078
ENSG00000076053	*RBM7*	1.148	0.190	0.728	0.200
ENSG00000244462	*RBM12*	5.530	0.028	4.575	0.017
ENSG00000239306	*RBM14*	0.882	0.028	1.247	0.192
ENSG00000147274	*RBMX*	5.781	0.011	4.702	0.017
ENSG00000020633	*RUNX3*	ND	ND	ND	ND
ENSG00000116560	*SFPQ*	6.759	0.009	5.362	0.014
ENSG00000188529	*SRSF10*	3.181	0.149	3.040	0.054
ENSG00000184402	*SS18L1*	0.916	0.317	0.856	0.212
ENSG00000172660	***TAF15***	3.400	0.060	3.245	0.001
ENSG00000143569	*UBAP2L*	6.232	0.001	5.205	0.032
ENSG00000188177	*ZC3H6*	2.827	0.066	2.762	0.195
ENSG00000198026	*ZNF335*	0.811	0.110	0.143	0.245
ENSG00000120948	*TARDBP*	4.411	0.028	3.083	0.097
		**Oocytes from small antral follicles**			
**Ensemble**	**Symbol**	**FPKM mean**	***t*****-test (*****p*****-value)**		
ENSG00000126705	*AHDC1*	0.416	0.5		
ENSG00000011243	*AKAP8L*	ND	ND		
ENSG00000140488	*CELF6*	ND	ND		
ENSG00000099622	*CIRBP*	2.916	0.444		
ENSG00000111605	*CPSF6*	0.871	0.272		
ENSG00000149532	*CPSF7*	2.361	0.429		
ENSG00000071626	*DAZAP1*	0.294	0.5		
ENSG00000064195	*DLX3*	ND	ND		
ENSG00000182944	***EWS/EWSR1***	8.638	0.149		
ENSG00000119812	*FAM98A*	0.307	0.320		
ENSG00000182263	*FIGN*	0.238	0.5		
ENSG00000089280	***FUS***	4.790	0.253		
ENSG00000139675	*HNRNPA1L2*	0.190	0.5		
ENSG00000169813	*HNRNPF*	5.995	0.232		
ENSG00000169045	*HNRNPH1*	4.243	0.050		
ENSG00000096746	*HNRNPH3*	4.087	0.375		
ENSG00000165119	*HNRNPK*	3.696	0.359		
ENSG00000125944	*HNRNPR*	6.266	0.169		
ENSG00000105323	*HNRNPUL1*	2.232	0.424		
ENSG00000176624	*MEX3C*	3.173	0.5		
ENSG00000147140	*NONO*	3.516	0.381		
ENSG00000167005	*NUDT21*	3.383	0.452		
ENSG00000121390	*PSPC1*	2.223	0.5		
ENSG00000102317	*RBM3*	0.069	0.5		
ENSG00000268489	*RBM3*	ND	ND		
ENSG00000173914	*RBM4B*	2.100	0.5		
ENSG00000076053	*RBM7*	ND	ND		
ENSG00000244462	*RBM12*	4.552	0.436		
ENSG00000239306	*RBM14*	0.069	0.5		
ENSG00000147274	*RBMX*	3.804	0.258		
ENSG00000020633	*RUNX3*	ND	ND		
ENSG00000116560	*SFPQ*	5.167	0.116		
ENSG00000188529	*SRSF10*	3.538	0.5		
ENSG00000184402	*SS18L1*	ND	ND		
ENSG00000172660	*TAF15*	2.933	0.442		
ENSG00000143569	*UBAP2L*	3.791	0.457		
ENSG00000188177	*ZC3H6*	4.146	0.189		
ENSG00000198026	*ZNF335*	0.069	0.5		
ENSG00000120948	*TARDBP*	5.918	0.025		

*1 *Genes alphabetically sorted*.

**Figure 2:**
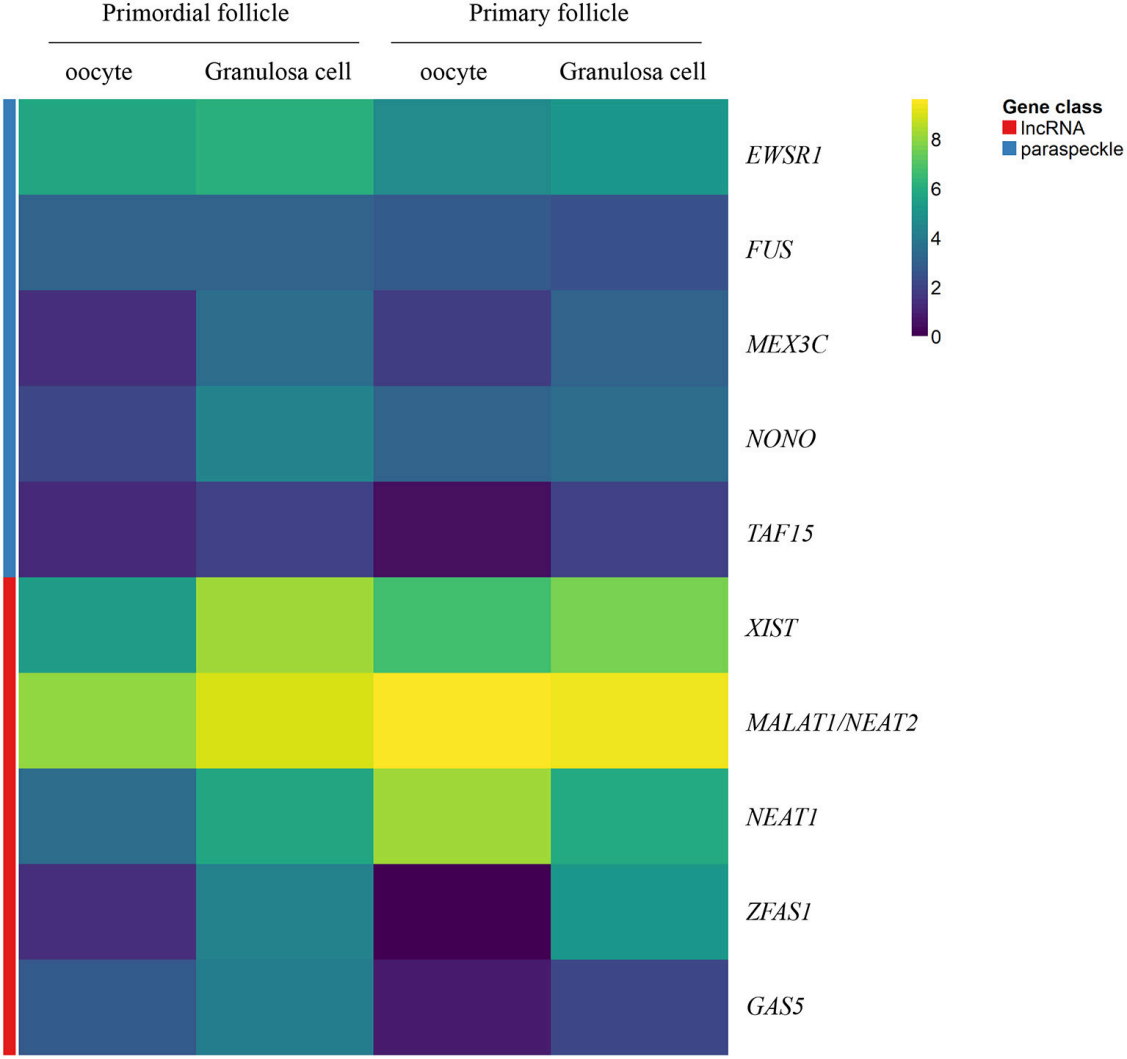
Heatmap representation of selected genes encoding paraspeckle proteins and lncRNA genes. Heatmap of gene expression in oocytes and granulosa cells during the primordial-to-primary follicle transition. The heatmap includes the paraspeckle-encoding genes (blue bar) *MEX3C, NONO*, FUS*, EWS, TAF15* transcripts, and the lncRNAs (red bar) *NEAT1, MALAT1* (*NEAT2*), *XIST, ZFAS1, GAS5*. Color code reflects average FPKM values.

*2 *Genes presented in Heatmap (Figure 2)*.

**Figure 3:**
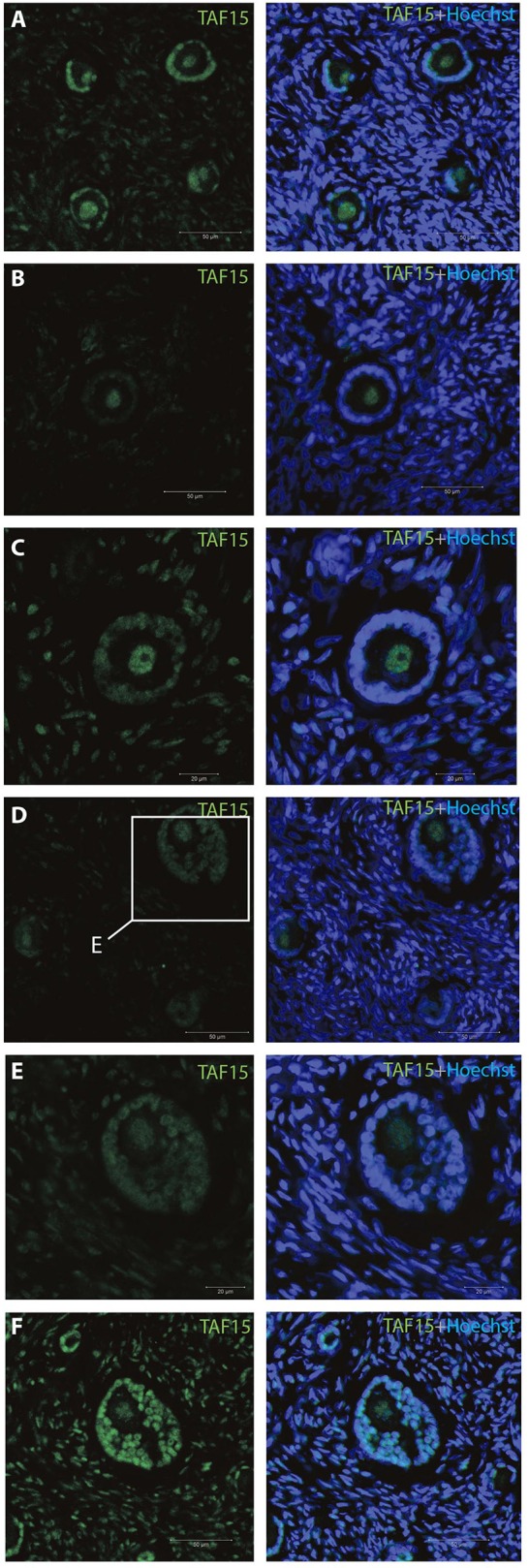
Intra-ovarian distribution of TAF15 in human primordial and primary follicles. This showed detection of the TAF15 protein in both oocyte and granulosa cells of **(A)** primordial, **(B)** primary, and **(C)** secondary, as well as **(D–F)** small pre-antral/early antral follicles. Hoechst staining identifies the nucleus of cells. Scale bars; 30 μm.

**Figure 4:**
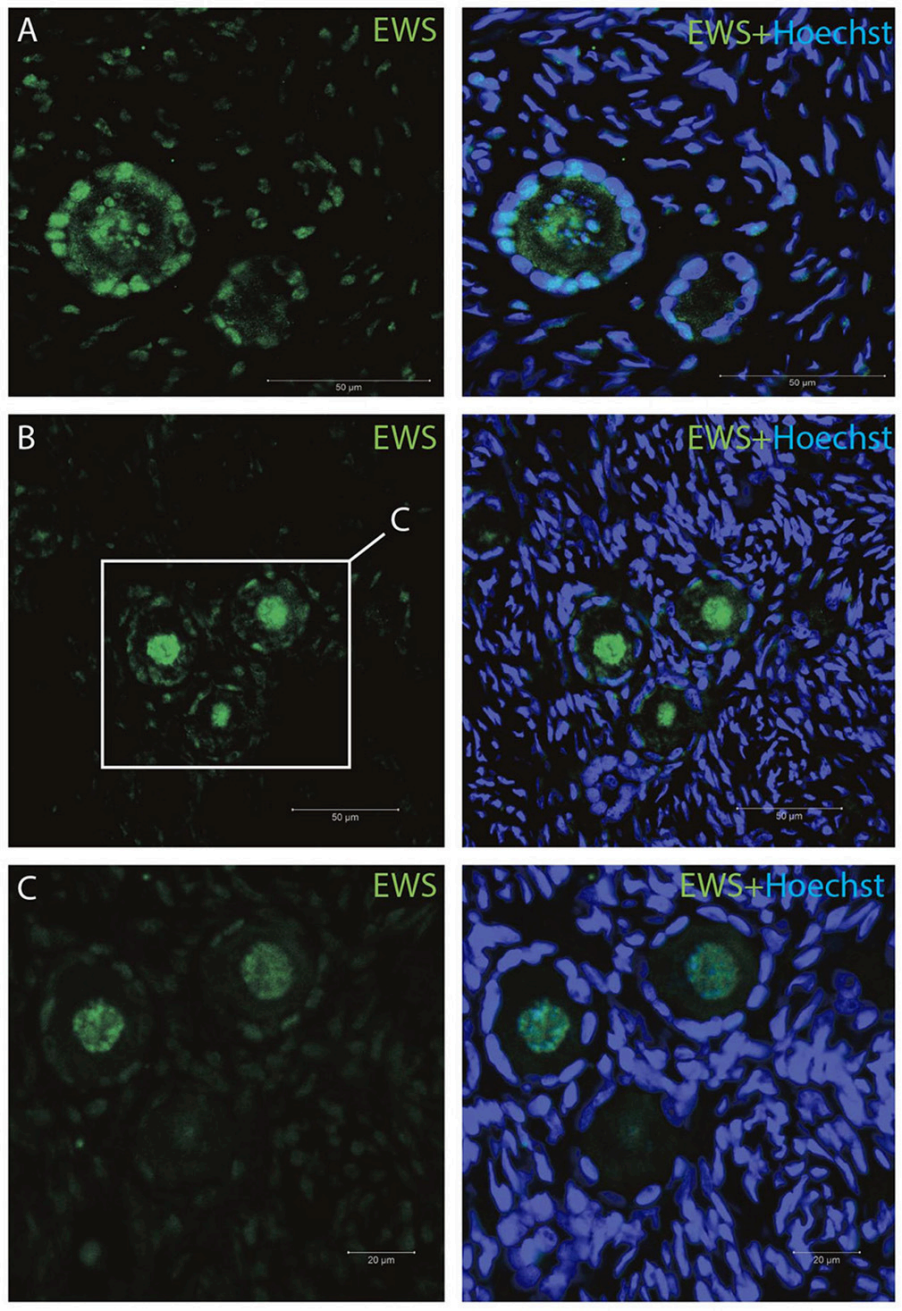
Intra-ovarian distribution of EWS in human primordial and primary follicles **(A-C)**. EWS is present in both oocytes and the surrounding granulosa cells in primordial and primary follicles. Hoechst staining identifies the nucleus of cells. Scale bars; 30 μm.

**Figure 5:**
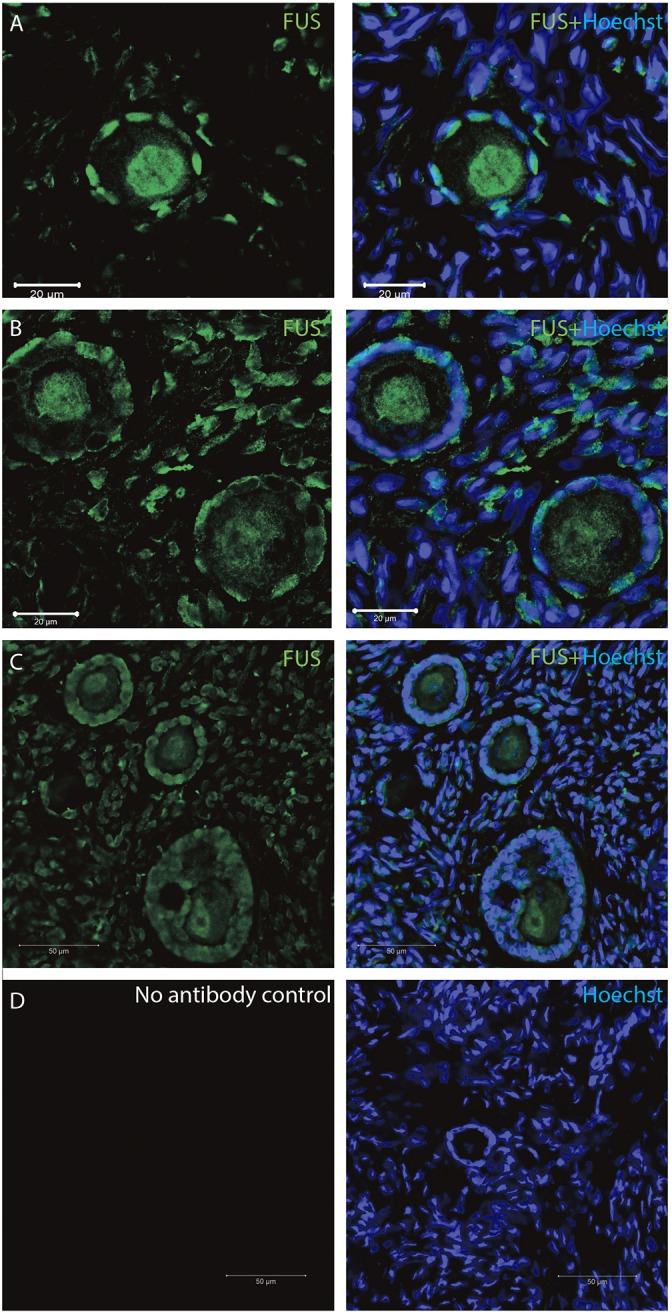
Intra-ovarian distribution of FUS in human primordial, and primary follicles. The FUS protein was detectable in **(A)** primordial follicles, **(B,C)** primary follicles **(C)** late pre-antral/early antral follicles. All samples were compared to a **(D)** no-antibody control. Hoechst staining identifies the nucleus of cells. Scale bars; 30 μm.

*3 *Transcripts in bold are used in immunofluorescence (Figures 3–5)*.

**Table 3:** Long non-coding RNAs (*p* < 0.05) in human follicle development.

**Oocytes from primordial follicles**			**Primordial follicle**		
Symbol^*1^	**FPKM mean**	***t*-test (*p*-value)**	**Symbol**	**FPKM mean**	***t*-test (*p*-value)**
**PRIMORDIAL FOLLICLES**					
ADCY10P1	0.763	0.046	BDNF-AS	2.395	0.017
LINC00485	2.7643	0.046	FGD5-AS1	3.335	0.013
LINC00924	0.171	0.015	GLG1	2.640	0.007
LINC01128	5.889	0.009	LINC00221	3.800	0.012
LINC01511	6.533	0.019	LINC00485	2.770	0.007
LOC100129434	6.859	0.0036	LINC00707	4.801	0.041
LOC100507557	3.783	0.005	LINC01483	2.976	0.040
LOC101927487	4.044	0.036	LOC100129434	6.765	0.012
LOC101928137	2.656	0.029	LOC100506885	3.548	0.028
LOC101929128	4.250	0.023	LOC100507156	2.776	0.013
LOC101929567	4.773	0.014	LOC100507557	2.443	0.018
LOC101929612	3.972	0.005	LOC101926943	1.428	0.008
LOC102467226	0.335	0.016	LOC101927487	3.216	0.035
LOC284798	2.494	0.002	LOC101928137	1.999	0.012
MALAT1	7.954	0.006	LOC101929567	3.513	0.033
MIR3609	5.490	0.012	LOC101929612	3.197	0.005
MIR99AHG	0.262	0.016	LOC440300	1.490	0.041
NPY6R	0.211	0.013	LOC643201	4.491	0.034
OIP5-AS1	4.190	0.029	MALAT1	8.992	0.026
RN7SK	11.285	0.005	MGC32805	0.219	0.039
RN7SL2	8.794	0.012	MIR4426	0.622	0.018
RPS3A	1.279	0.021	OIP5-AS1	5.527	0.033
UGDH-AS1	6.637	0.013	RN7SK	11.227	0.001
XIST	5.393	0.026	RN7SL2	8.9514	0.001
			RPL13AP5	2.322	0.002
			RPL21P28	0.582	0.012
			RPS3A	1.118	0.043
			SCARNA7	5.888	0.039
			SLC8A1-AS1	5.409	0.007
			SYN2	4.108	0.043
			TUNAR	3.893	0.047
			UBXN8	2.491	0.050
			UGDH-AS1	6.382	0.006
			XIST	8.236	0.003
			ZFAS1	4.296	0.014
			ZNF252P	3.537	0.038
**Oocytes from primary follciles**			**Primary follicle**		
**Symbol**	**FPKM mean**	***t*****-test (*****p*****-value)**	**Symbol**	**FPKM mean**	***t*****-test (*****p*****-value)**
**PRIMARY FOLLICLES**					
BCAR4	4.563	0.008	CEACAM22P	0.182	0.033
LINC00485	1.902	0.002	GLG1	1.270	0.046
LINC00665	4.298	0.046	KIZ	4.874	0.014
LINC01511	4.625	0.016	LINC01511	5.425	0.028
LOC100129434	6.444	0.011	LOC100129434	5.792	0.047
LOC100506885	2.187	0.039	LOC100507557	1.854	0.041
LOC101926943	1.548	0.044	LOC101927487	3.822	0.007
LOC101927337	2.226	0.014	LOC101929567	4.053	0.049
LOC101929491	0.330	0.004	LOC101929612	3.456	0.010
LOC101929567	4.287	0.008	MALAT1	9.345	0.016
LOC101929612	3.152	0.016	NEXN-AS1	0.534	0.046
LOC102546299	2.551	0.001	OIP5-AS1	5.950	0.017
MALAT1	9.614	0.001	RN7SK	11.308	0.004
MEG3	0.220	0.024	RN7SL2	8.743	0.001
MIR3609	3.887	0.012	RPL13AP5	2.877	0.003
MIR4426	0.595	0.020	RPL21P28	0.868	0.008
RN7SK	11.845	0.001	RPL21P28	2.165	0.032
RN7SL2	9.110	0.004	SNORD89	1.513	0.034
RPL13AP5	1.418	0.011	UGDH-AS1	6.216	0.016
RPL21P28	0.916	0.047	XIST	7.610	0.030
RPS3A	0.894	0.007	ZFAS1	5.249	0.005
SCARNA7	5.424	0.009			
UGDH-AS1	6.397	0.003			
XIST	6.751	0.014			
ZNF518A	2.983	0.018			
**Mural granulosa cell**			**Theca cells**		
**Symbol**	**FPKM mean**	***t*****-test (*****p*****-value)**	**Symbol**	**FPKM mean**	***t*****-test (*****p*****-value)**
**SMALL ANTRAL**					
ANP32AP1	0.683	0.009	ANKRD36B	2.277	0.020
CASP8AP2	3.530	0.034	BCYRN1	2.661	0.034
CROCCP2	1.453	0.006	BDNF-AS	2.317	0.033
EBLN3	3.812	0.002	CD27-AS1	2.007	0.041
FGD5-AS1	4.032	0.019	CLEC2D	1.283	0.027
GOLGA6L5P	1.750	0.015	CSNK1A1	2.753	0.001
H3F3AP4	2.121	0.027	CTBP1-AS2	2.457	0.028
LINC00657	5.467	0.009	FGD5-AS1	3.506	0.045
LINC01128	4.413	0.018	FLJ42627	0.239	0.000
LINC01420	2.152	0.012	H3F3AP4	2.185	0.014
LOC100129434	3.831	0.025	HCG18	3.976	0.035
LOC100131564	2.425	0.024	HERC2P3	0.111	0.017
LOC100507557	1.904	0.028	LINC00485	1.467	0.049
LOC101927027	1.875	0.003	LINC00657	4.740	0.009
LOC101929124	3.0195	0.010	LINC01128	3.643	0.020
LOC101929612	3.418	0.007	LINC01133	0.111	0.017
LOC102477328	0.135	0.020	LOC100129434	4.893	0.008
LOC150776	3.000	0.002	LOC101929612	3.455	0.001
LOC643201	3.740	0.013	LOC102724699	0.420	0.021
LOC646762	2.431	0.008	MAGI2-AS3	2.791	0.020
LOC728554	1.619	0.023	MALAT1	10.73	0.000
MALAT1	9.495	0.006	MEG3	7.602	0.001
MIR3609	5.033	0.001	MIR3609	4.974	0.009
MIR99AHG	3.823	0.014	MIR4426	0.504	0.043
OIP5-AS1	5.926	0.003	MIR99AHG	5.083	0.008
PCBP1-AS1	2.541	0.029	NCBP2-AS2	0.824	0.001
PIGBOS1	1.804	0.011	NEXN-AS1	0.735	0.025
PKI55	4.600	0.000	NKAPP1	3.545	0.032
RN7SK	10.395	0.002	OIP5-AS1	4.636	0.039
RN7SL2	8.088	0.004	PCBP1-AS1	4.558	0.009
RPL13AP5	2.509	0.006	PDIA3P1	0.450	0.016
RPL21P28	2.293	0.012	PGM5P2	1.302	0.028
RPL21P28	0.589	0.026	PKI55	4.791	0.024
RPL34P6	0.405	0.013	PTOV1-AS1	2.060	0.001
RPS3A	2.326	0.001	RN7SK	10.57	0.005
SCAND2P	1.312	0.014	RN7SL2	8.740	0.006
SCARNA7	5.104	0.016	RNU4-2	4.075	0.007
SNHG17	3.099	0.018	RPL13AP5	2.816	0.011
TMEM120A	3.042	0.002	RPL21P28	2.840	0.006
TUG1	6.0234	0.003	RPL21P28	1.081	0.017
UGDH-AS1	3.874	0.031	RPL34P6	0.844	0.019
XIST	8.056	0.004	RPS3A	1.846	0.010
ZFAS1	5.124	0.005	SCARNA7	5.588	0.026
ZNF761	4.206	0.017	SDHAP2	2.223	0.001
ZNF826P	0.990	0.018	SH3BP5-AS1	2.069	0.008
			SNORA23	0.659	0.007
			SNORA79	1.713	0.039
			SNORD89	2.354	0.017
			SPON1	3.979	0.030
			THUMPD3-AS1	0.829	0.049
			TMEM120A	1.996	0.042
			TUG1	6.154	0.008
			UBXN8	4.509	0.006
			UGDH-AS1	4.596	0.000
			XIST	8.474	0.001
			ZFAS1	4.881	0.013
			ZNF252P	1.822	0.012
			ZNF518A	2.901	0.020
			ZSCAN26	3.800	0.007
**Oocyte from small antral follicle**					
**Symbol**	**FPKM mean**	***t*****-test (*****p*****-value)**			
LOC100653061	5.033	0.033			
MALAT1	7.219	0.027			
OIP5-AS1	0.842	0.007			
PMS2CL	0.481	0.008			
RN7SK	10.140	0.021			
ROR1-AS1	0.481	0.008			

*1 *Genes alphabetically sorted*.

^*2^*Presented in heat map (Figure 2)*.

### Immunofluorescence microscopy

Human ovarian cortical tissue was cut in 5 μm sections and mounted on glass slides. Dehydration and antigen retrieval was performed as described elsewhere (Stubbs et al., 2005) followed by serum block (30 min), then the primary antibody; (1/200) anti-TAFII68 Rabbit pAb (Bethyl Laboratories, #IHC-00094), (1/500) anti-FUS Rabbit pAb (Bethyl Laboratories, #A300-302A), or (1/200) anti-EWS Rabbit pAb (Bethyl Laboratories, #IHC-00086) overnight at 4°C. The sections were then incubated in a 1:700 dilution of secondary antibody (Donkey-anti-Rabbit) conjugated with Alexa Fluor 488 Dye (Life Technologies). Finally, sections were incubated in 1/7500 Hoechst (Life Technologies) followed by mounting with Dako Fluorescent Mounting Medium (Agilent Technologies, Santa Clara, CA, U.S.A.) and analyzed using a LSM510 laser-scanning confocal microscope using a 63x C-Apochromat water immersion objective NA 1.2 (Carl Zeiss, Göttingen, Germany) and ZEN 2011 software (Carl Zeiss, Göttingen, Germany).

## Results

### Laser-isolation of oocytes and somatic cells during human follicle development

Specific isolates of oocytes and follicles (oocytes with surrounding somatic granulosa cells) from the primordial and primary stage, respectively, as well as oocytes, mural granulosa cells, and theca cells from small antral follicles were collected via Laser Capture Microdissection (LCM). Each stage was isolated on the basis of stringent morphological criteria (Gougeon, 1996) (Figure 1). Primordial follicles were defined as an oocyte surrounded by one layer of flattened granulosa cells (Figure 1) and primary follicles were defined as an oocyte surrounded by a single layer of cubic granulosa cells (Figure 1). Small antral follicles were defined based on the presence of a follicular antrum with a clear distinction between the oocyte, the mural granulosa cells and the theca cell layer (Figure 1) The samples (1,473 isolates) of cells from primordial, primary, and small antral follicles were pooled into 20 samples (Table 1, Figure 1). These 20 samples were then subjected to RNA sequencing using the IlluminaHiSeq2000 sequencing platform (Illumina Inc., San Diego, CA, U.S.A.) at an external sequencing facility (AROS Applied Biotechnology, Aarhus, Denmark). We previously validated the expression pattern for various RNAs in the present RNA seq. dataset using RT-qPCR (Ernst et al., 2017, 2018). The RNA sequencing yielded on average 35.3 million reads per sample (range: 31.8–39.6 million reads) and was mapped to the human genome (hg19) (average number of reads mapped: 31.7 million, range: 29.4–34.0). Gene expression was calculated as FPKM by a custom R script (Ernst et al., 2017, 2018).

### Transcriptional profiles of genes encoding paraspeckle proteins across different follicle stages

The expression of 39 genes encoding paraspeckle proteins (Naganuma et al., 2012) was interrogated during human follicle development (Table 2). The highest expression of paraspeckle genes in the primordial follicle stage, based on FPKM values, were *EWS, HNRNPK, ZC3H6, UBAP2L*, and *TARDBP* (Table 2). Several other genes encoding paraspeckle proteins were present (e.g., *MEX3C, FUS, TAF15, CPSF6, NUDT21, RBM12, RBMX, DLX3*).

Interestingly, the expression of *ZC3H6* appears to be downregulated from primordial to primary follicles, indicating a specific function associated with the primordial follicle. The *EXSR1* gene expression remains high and upregulated in small antral follicles. *NONO* was noted to be upregulated as follicle development advances, with the highest expression detected in the somatic cells in the small antral follicle (Table 2).

A heatmap of FPKM data for selected genes encoding paraspeckle genes was generated to show the expression for the two different cell-stages in isolates - and the correlation between cell-specific isolates (Figure 2).

### Intra-ovarian distribution of paraspeckle proteins TAF15, EWS, and FUS

The gene products of *TAF15, EWS*, and *FUS* were selected for immunofluorescent staining (IMF) (bold in Table 2) to reveal their localization in human ovarian sections.

The TAF15 translational product was expressed in both oocytes and follicles from primordial, primary, and small antral stages, with a particular high expression in oocytes from primordial follicles, as well as in primordial follicles (Table 2). We interrogated the TAF15 protein using a specific antibody toward TAF15. This showed detection of the TAF15 protein in both oocyte and granulosa cells of primordial (Figure 3A), primary (Figure 3B), secondary (Figure 3C), as well as small pre-antral/early antral follicles (Figures 3D–F). As the TAF15 protein appears detectable in both oocytes and the surrounding somatic cells are in line with the RNA sequencing data, gene expression and its translational product appears coupled.

The IMF of EWS showed that EWS is present in both oocytes and the surrounding granulosa cells, and in primordial and primary follicles (Figures 4A–C). The *EWS* transcript was found highly expressed in these early stages of follicle development, and thus the RNA expression appears coupled to its translational protein product.

The *FUS* transcript was also highly expressed during early follicle development (Table 2), and as we interrogated its protein using IMF, found that the FUS protein was detectable in primordial follicles, (Figure 5A), primary follicles (Figures 5B, C), as well as in late pre-antral/early antral follicles (Figure 5C).

All samples were compared to a no-antibody control, which did not detect any signal (Figure 5D).

We detected nuclear localization of TAF15, EWS and FUS, with evidence of speckle-like structures in an infrequent manner distributed throughout the cells.

### Non-coding RNAs

ncRNA genes produce functional RNA molecules rather than encoding proteins (Eddy, 2001). The groups of ncRNA are diverse and include, for instance, short and long ncRNAs as well as micro (mi)RNA, snoRNA, scaRNA, SRP RNA, and antisense RNA. The presence of ncRNAs during human follicle development was analyzed in transcriptomes representative for oocytes and follicles from primordial and primary follicles as well as oocytes, mural granulosa cell layers, and theca cell layers of small antral follicles [Figure 1, Table 1, (Resource Data)]. Genes encoding ncRNAs was identified using the Ensembl gene annotation version GRCh37.p13.

### Long ncRNAs

Most ncRNAs longer than 200 nucleotides are referred to as ‘long non-coding RNAs’ (lncRNAs). Although the estimated number of different types of human lncRNAs has ranged from 5,400 to 53,000 (Palazzo and Lee, 2015), these ncRNAs appear to comprise functions for the control of various levels of gene expression in physiology and development, including chromatin architecture/epigenetic memory, transcription, RNA splicing, editing, translation, and turnover (Mattick and Makunin, 2006). In this study the presence of lncRNAs was interrogated (Table 3).

Interestingly, we detected the lncRNA *XIST* during human follicle development in both oocytes and follicle samples (Table 3). It should be noted, however, that *XIST* in the oocytes from small antral follicles did not display a cell-specific consistently expressed expression pattern but was noted used a less stringent *p*-value (Resource Data), which was likewise observed for *TSIX* (Table 3). The fact that a less stringent *p*-value was needed to detect this transcript in the oocytes from small antral follicles was expected, as these oocytes were rarely found in the ovarian biopsies, and thus these oocyte samples are less represented (Table 1, Figure 1).

*MALAT1 (NEAT2)* was detected throughout all the included stages (Resource Data), indicating the need for this paraspeckle-forming protein during human follicle development. Interestingly, several lncRNAs with no biological functions annotated were noted (Resource Data). While the expression of several lncRNA genes (*OIP5-AS1, RN7SK, RN7SL2*) was present in all samples tested, others appeared to be cell- and stage-specifically (*GLG1, KIZ, BCAR4, EBLN3*) expressed (Resource Data).

The lncRNA *ZFAS1* appears to be restricted to somatic cells, e.g., the mural granuloma cell layer and the theca cell layer in the small antral follicle (Resource Data). Interestingly, the *ROR1-AS1* seems to be specific to the oocyte from small antral follicles (Resource Data**)**. We found lncRNA Growth Arrest Specific 5 (*GAS5*) expressed in oocytes from primordial follicles, as well as a high expression in primordial follicles, somewhat lower in primary follicles, and in turn high in the mural granulosa cell layer and the theca cell layer from small antral follicles (Resource Data).

A heatmap of FPKM data for selected lncRNA genes was generated to show the expression for the two different cell-stages (primordial versus primary) in isolates - and the correlation between cell-specific (oocyte versus granulosa cell) isolates (Figure 2).

## Discussion

Extensive efforts to gain deeper understanding of RNA biology have yielded evidence of the diverse structural and regulatory roles in protecting chromosome integrity, maintaining genomic architecture, X chromosome inactivation, imprinting, transcription, translation and epigenetic regulation (Khorkova et al., 2015). Bioinformatics analysis of chromatin marks in intergenic DNA regions and of expressed sequence tags (ESTs) predicts the existence of more than 5,000 long noncoding RNA (lncRNA) genes in the human genome (Gomez et al., 2013). Some studies have found the number of lncRNAs to exceed that of protein-coding genes (Bouckenheimer et al., 2016; Hon et al., 2017). In our transcriptome study of lncRNA, we applied a strict filter to only consider transcript that were consistently expressed in our samples. This was applied as a major limiting factor is the number of patients included in the study. In oocytes and granulosa cells from primordial and primary follicles, 20, 33, and 20 and 19 lncRNAs were noted expressed (using a cut of value of 1 FPKM). Interestingly comparing this to the number of protein coding transcripts in the same stages (oocytes and granulosa cells from primordial and primary follicles showed 1099, 1695, and 1046 and 815, SSCEG, respectively (Ernst et al., 2017, 2018), it is noteworthy that few lncRNAs compared to the protein coding transcript are present during these early stages in human follicle development.

Strict filters in the bioinformatic management was applied to this study to ensure the most precise outcome from the global transcriptome analysis. Previous studies validated selected candidates by qPCR analysis (Ernst et al., 2017, 2018). Moreover, the analysis contains several DEG-lists based on both SSCEGs and non-SSCEGs and caution in the analysis of fold of change for DEG transcripts is recommended. Importantly, this study analyzed the presence of transcripts, and whether a gene is translated or its protein product present, is unknown. Using single cell techniques, we confirmed the presence of selected paraspeckle proteins using immunohistochemistry.

In a few well-studied cases, such as *AIR, XIST*, and *HOTAIR*, these lncRNAs have been shown to operate at the transcriptional level by binding to proteins in histone-modifying complexes and targeting them to particular genes (Nagano et al., 2008; Chu et al., 2011; Jeon and Lee, 2011; Wang and Chang, 2011). A role for lncRNAs in human follicle development has not previously been described (Wilhelmm and Bernard, 2016) although their potential involvement has been suggested (Zhao and Rajkovic, 2008; Bouckenheimer et al., 2016). Differential lncRNA expression profiles in human oocytes and cumulus cells was recently analyzed (Bouckenheimer et al., 2018), which determined the lncRNA expression profiles of human MII oocytes (*BCAR4, C3orf56, TUNAR, OOEP-AS1, CASC18*, and *LINC01118*) and cumulus cells (*NEAT1, MALAT1, ANXA2P2, MEG3, IL6STP1*, and *VIM-AS1*).

The presence of the paraspeckle-forming *NEAT1* and *MALAT1* (*NEAT2*) indicates that paraspeckles are actively formed and present during human follicle development. Paraspeckle formation is initiated by transcription of the *NEAT1* chromosomal locus and proceeds in conjunction with *NEAT1* lncRNA biogenesis and a subsequent assembly step involving >39 paraspeckle proteins (PSPs). Interestingly, a study has shown that subunits of SWItch/Sucrose NonFermentable (SWI/SNF) chromatin-remodeling complexes were identified as paraspeckle components that interact with PSPs and *NEAT1* lncRNA (Kawaguchi et al., 2015). In particular, it was shown by electron microscopy that SWI/SNF complexes were enriched in paraspeckle subdomains depleted of chromatin. Interestingly, and consistent with this, it was found that the arginine methyltransferase CARM1 (coactivator-associated arginine methyltransferase 1) promotes the nuclear export of mRNAs that contain inverted Alu elements in their 3' untranslated region by methylating the paraspeckle component p54(nrb), which reduces the binding of p54(nrb) to the inverted Alu elements. It also down-regulated the synthesis of NEAT1. This in turn inhibited paraspeckle formation (Elbarbary and Maquat, 2015).

The lncRNA *XIST* was present at high levels throughout the stages tested during human follicle development. To ensure X-linked gene dosage compensation between females (XX) and males (XY), one X chromosome randomly undergoes X chromosome inactivation (XCI) in female cells (Lyon, 1961). The human *XIST* (Brown et al., 1991a,b) and mouse *Xist* (Borsani et al., 1991; Brockdorff et al., 1991) lncRNAs accumulate over the X chromosome. X chromosomal inactivation is tightly regulated throughout development with *XIST* as a key regulator involved in the establishment of several layers of repressive epigenetic modifications. These reported functions of *XIST* are consistent with our observation that this gene is highly transcribed during human follicle development and reveals that *XIST* lncRNA is present already from the dormant primordial stage of human follicle development. The functional role of *XIST* during early follicle development remains to be elucidated, and this may include early marks of maternal imprinting and dosage regulation.

The lncRNA *ZFAS1* appears specific to somatic cells during human follicle development. *ZFAS1* has been described as being upregulated in different cancer types (Askarian-Amiri et al., 2011; Li et al., 2015; Nie et al., 2016; Thorenoor et al., 2016) and is involved in cell apoptosis and cell cycle control. It was recently shown that the action of *ZFAS1* occurred through interaction with EZH2 and LSD1/CoREST in order to repress the underlying targets KLF2 and NKD2 transcription (Nie et al., 2016). The epigenetic dysregulation of central granulosa cell factors such as FOXL2 are involved in the development of granulosa cell tumors (Xu et al., 2016), which is possible through EZH2 interaction. Furthermore, prominent roles for FOXL2 include control of primordial follicle activation (Schmidt et al., 2004).

It remains to be tested if lncRNA *ZFAS1* functions to regulate transcriptional control in follicle development, and this may have an effect on granulosa cell proliferation and cell cycle control in the human follicle.

We identified the potential tumor suppressor lncRNA growth arrest specific 5 (*GAS5*), expressed particularly in the primordial stage, as well as in primary follicles and in mural granulosa cell layers and the theca cell layers. Part of the *GAS5* RNA structure mimics the glucocorticoid response element, enabling it to bind the DNA binding domain of the glucocorticoid receptor, thus inhibiting glucocorticoid induced transcription. *GAS5* is further thought to regulate transcriptional activity of the androgen receptor. In line with this, *GAS5* lncRNA has been found to repress the AR/androgen complex from binding to target through sequestering, thus repressing transcription (Wang and Lee, 2009). Of further interest, *GAS5* lncRNA has been found to supress the AKT/mTOR signaling pathway in prostate cancer cells (Yacqub-Usman et al., 2015). As previous studies have shown that activated AKT/mTOR signaling increases primordial follicle activation (Makker et al., 2014), we suggest that *GAS5* expression in the primordial follicle may be involved in primordial follicle dormancy and survival. Recently, the GAS5 was found to promote proliferation and survival of female germline stem cells *in vitro* (Wang et al., 2018). The functional involvement of *GAS5* in normal and aberrant human follicle development remains to be determined.

Increasing evidence supports a central role for ncRNA in numerous aspects of chromatin function (Názer and Lei, 2014). Interestingly, it has long been appreciated that ncRNAs are central components of the dosage compensation machinery, and recent work has elucidated how various ncRNAs contribute to Polycomb Group (PcG) and chromatin insulator activities (reviewed in Názer and Lei, 2014). The PcG proteins are required for the adequate development of multicellular organisms, functioning to preserve pluripotency and/or cellular identity. Their main function however is to repress the expression of genes that would otherwise promote differentiation into other cell types (reviewed in Simon and Kingston, 2013).

The precise role of lncRNAs in chromatin modifications during human follicle development remains to be elucidated. However, our data suggests that several distinct lncRNAs are present and that they probably have separate functions in order to secure follicle integrity and development.

We found the protein-coding *MEX3C* gene present during human follicle development. However, the role of the gene product, MEX3C, is unknown. The MEX3BM isoform and the E3 ubiquitin ligase DZIP3 are bought together with their substrates (Ataxin-1 and Snurportin-1) by the lncRNA *HOTAIR*, accelerating their degradation (Khorkova et al., 2015), thus lncRNA-mediated regulation also affects protein stability (reviewed in Khorkova et al., 2015). Furthermore, proteasomal inhibition causes upregulation of paraspeckle-associated lncRNA *NEAT1*, which in turn protects fibroblasts from cell death triggered by proteasome inhibition (Khorkova et al., 2015). Interestingly, it was found that a MEX3 homolog is required for differentiation during planarian stem cell lineage development (Zhu et al., 2015). In this study it was shown that MEX3-1 was required for generating differentiated cells of multiple lineages, while restricting the size of the stem cell compartment. This indicates that MEX3-1 functions as a cell fate regulator (Zhu et al., 2015). The presence of the *MEX3C* transcript during human follicle development has not been functionally addressed, and future studies should reveal whether MEX3C has a pivotal role in cell commitment and/or differentiation in the selection of the dominant follicle.

The upregulated expression profile of *NONO* during follicle development suggests that this protein is under tight control. NONO deficiency led to upregulation of PSPC1, which replaces NONO in a stable complex with SFPQ (Li et al., 2014). The knockdown of PSPC1 in a *Nono*-deficient background led to severe radio-sensitivity and delayed resolution of double stranded break (DSB) repair foci. From this it can be concluded that NONO or related proteins are critical for DSB repair (Li et al., 2014). The complex of NONO with SFPQ and PSPC1 served a multipurpose scaffold, including frequently identified engagement at almost every step of gene regulation, and including, but not limited to, transcriptional regulation, RNA processing and transport, and DNA repair (Knott et al., 2016). Interestingly, a report has investigated the inner cell mass marker OCT4 and its gene expression patterns, as well as CpG sites methylation profiles during embryonic stem (ES) cell differentiation into neurons (Park et al., 2013). It was found that NONO binds to the CpG island of the *Oct4* promoter and positively regulates *Oct4* gene expression in ES cells (Park et al., 2013), thus indicating a role in cell lineage during early development. The future role of NONO during human follicle development and how this might participate in regulating gene expression and/or DNA repair will be important steps toward the understanding of the capacity of the human ovarian follicles.

Several lines of evidence suggest paraspeckle proteins to be essential in cell fate determination, which is highly relevant for early developmental processes (Yamazaki and Hirose, 2015).

FUS, EWS, and TAF15 are structurally similar multifunctional proteins that were initially discovered in the process of characterization of fusion oncogenes in human sarcomas and leukemias. As they are implicated in numerous central cellular processes such as gene regulation, genomic integrity maintenance and mRNA/microRNA processing, it is therefore not surprising to find them in many cellular contexts and in different cell types and tissues. The expression profile of the FET proteins were characterized in both the human (Andersson et al., 2008) and porcine (Blechingberg et al., 2012b) developing brain. The FET proteins are expressed in most human tissues and are localized mainly in the cell nucleus (Andersson et al., 2008), but are also found in the cytoplasm (Zinszner et al., 1997; Belyanskaya et al., 2001; Jobert et al., 2009). This is supported by the fact that the functions of hnRNPs include nucleocytoplasmic shuttling (Bedford and Clarke, 2009; Yu, 2011). Interestingly, FUS, EWS, and TAF15 has previously revealed a cell-specific expression pattern (Andersson et al., 2008), and processes such a heat shock and/or oxidative stress induce the re-localization of these proteins to stress granules (Andersson et al., 2008; Blechingberg et al., 2012a). The fact that we observed infrequent staining in nuclear and cytoplasmic localizations supports the activity of these FET proteins.

The FET proteins also frequently exhibit gene translocation in human cancers (Paronetto, 2013; Campos-Melo et al., 2014). Emerging evidence demonstrates their physical interactions with DNA Damage Response proteins (Kai, 2016) and thus suggests their involvement in the maintenance of genome stability. Interestingly, it was recently proposed that FET proteins are involved in the maintenance of lifespan, cellular stress resistance, and neuronal integrity (Therrien et al., 2016).

It has been shown that FUS interact directly with *NEAT1* lncRNA, reducing the expression of FUS, and subsequently causing cell apoptosis. In combination with miR-548ar-3p, this regulates breast cancer cell apoptosis (Ke et al., 2016).

## Conclusion

We identified the presence of lncRNAs as well as the genes encoding the paraspeckle proteins, offering insights into how their transcripts are expressed during human follicle development. The study is descriptive in nature. As a proof of concept, we probed for the intracellular presence and localization of three selected paraspeckle proteins. It remains to be determined for several other proteins encoding by genes noted, as well as lncRNAs. In particular, our study indicates that they may be involved in cellular processes such as cell differentiation and cell integrity. This could be accomplished by their ability to control gene expression, epigenetics and mRNA turnover during follicle development.

## Author contributions

EE and KL-H conceived the study. PV performed bioinformatics analysis. EE, JN, and MI performed IMF. EE and KL-H analyzed RNA sequencing data and wrote the manuscript. All authors approved the final manuscript.

### Conflict of interest statement

The authors declare that the research was conducted in the absence of any commercial or financial relationships that could be construed as a potential conflict of interest.

## Supplementary material

Supplementary Table 1 Full lists of ncRNAs detected in human follicle development [oocytes from primordial follicles (tab 1), primordial follicles (tab 2), oocytes from primary follicles (tab 3), mural granulosa cell layers from small antral follicles (tab 4), theca cell layers from small antral follicles (tab 5), oocytes from small antral follicles (tab 6)]. Cell specific consistently expressed genes (CSCEG) (one-sample *t*-test *p*-value < 0.05 in gray).
